# Neuroimaging Studies of Nonsuicidal Self-Injury in Youth: A Systematic Review

**DOI:** 10.3390/life11080729

**Published:** 2021-07-22

**Authors:** Marcelo J. A. A. Brañas, Marcos S. Croci, Ana Beatriz Ravagnani Salto, Victoria F. Doretto, Eduardo Martinho, Marcos Macedo, Euripedes C. Miguel, Leonardo Roever, Pedro M. Pan

**Affiliations:** 1 National Institute of Developmental Psychiatry for Children and Adolescents (INPD), São Paulo 05403-903, Brazil; marcelobranas@gmail.com (M.J.A.A.B.); marcoscroci@gmail.com (M.S.C.); ravagnani.beatriz@gmail.com (A.B.R.S.); dravictoriadoretto@gmail.com (V.F.D.); ecmiguel@usp.br (E.C.M.); leonardoroever@hotmail.com (L.R.); 2 Department of Psychiatry, Faculdade de Medicina, Universidade de São Paulo, São Paulo 05403-903, Brazil; eduardomartinhojr@gmail.com; 3 Laboratory of Integrative Neurosciences (LiNC), Universidade Federal de São Paulo, São Paulo 04021-001, Brazil; marcoscfmacedo@gmail.com

**Keywords:** systematic review, nonsuicidal self-injury, NSSI, self-harm, self-mutilation, youth, neuroimaging, fMRI, Newcastle–Ottawa Scale, JBI Critical Appraisal Checklist

## Abstract

Nonsuicidal self-injury (NSSI) is prevalent and affects mainly the youth population. It is prospectively associated with suicide attempts, making it a target for suicide prevention. Recently, several studies have investigated neural pathways of NSSI using neuroimaging. However, there is a lack of systematized appraisal of these findings. This systematic review aims to identify and summarize the main neuroimaging findings of NSSI in youth. We followed PRISMA statement guidelines and searched MEDLINE, APA PsycInfo, and Google Scholar databases for neuroimaging studies, irrespective of imaging modality, specifically investigating NSSI in samples with a mean age of up to 25 years old. Quality assessment was made using the Newcastle–Ottawa and Joanna Briggs Institute scales. The initial search retrieved 3030 articles; 21 met inclusion criteria, with a total of 938 subjects. Eighteen studies employed functional neuroimaging techniques such as resting-state and task-based fMRI (emotional, interpersonal exposure/social exclusion, pain, reward, and cognitive processing paradigms). Three studies reported on structural MRI. An association of NSSI behavior and altered emotional processing in cortico-limbic neurocircuitry was commonly reported. Additionally, alterations in potential circuits involving pain, reward, interpersonal, self-processing, and executive function control processes were identified. NSSI has complex and diverse neural underpinnings. Future longitudinal studies are needed to understand its developmental aspects better.

## 1. Introduction

Nonsuicidal self-injury (NSSI) refers to the direct and intentional self-inflicted lesion of body tissue without lethal intention or socially sanctioned purposes [1]. Although NSSI may occur in the absence of mental conditions, psychiatric disorders can be identified in up to 85% of the cases [2]. This condition has been historically linked to borderline personality disorder (BPD), but recent studies showed that NSSI is associated with several other mental conditions such as mood, anxiety, trauma, substance use, eating disorders, and even autism spectrum disorder [3,4]. Even though previous research has demonstrated the clinical relevance of NSSI, its underlying biological mechanisms remain poorly understood [5,6]. Here, we systematically review neuroimaging findings from NSSI studies conducted in the youth population. 

Recent classifications for mental health conditions, such as the DSM-5, recognize the relevance of NSSI and consider this behavior as a separate condition, independent of other psychiatric disorders but still requiring further studies [7,8]. In non-clinical samples, NSSI prevalence is 17.2% in adolescence and 13.4% in young adults [9], although rates vary widely among studies [10,11]. NSSI usually begins in early adolescence with a peak incidence around 15–17 years of age [12]. In youth, NSSI predicts suicidality [13], which is the second leading cause of death among adolescents [14]. Therefore, understanding the neural underpinnings of NSSI in youth, when this behavior emerges, can inform preventive strategies for suicide. Early structural and functional neuroimaging studies focused on NSSI among BPD patients. For instance, self-harm behavior was associated with increased pituitary volume in young BPD patients [15,16]. This finding suggested that NSSI behavior may be related to the hyper-reactivity of stress response systems [5,16]. In addition, new evidence emerged linking altered limbic processing, such as hyperactivity of the amygdala and anterior cingulate cortex (ACC), with NSSI [17].

Previous reviews have examined early neuroimaging findings for NSSI. Huang et al. (2020) found that self-injurious thoughts and behaviors were linked to hyperactivation of the right amygdala, left hippocampus, and left posterior cingulate cortex (PCC) [18]. These regions are responsible for mental processes implicated in NSSI, such as mentalization and emotional processing [18]. Auerbach et al. (2020) performed a comprehensive review that included 47 studies, including NSSI and suicidal thoughts and behaviors. Structural alterations among NSSI patients were reported, such as a volumetric reduction of the insula, ACC, and right inferior frontal gyrus. In addition, functional magnetic resonance imaging (fMRI) studies suggested frontolimbic aberrant activation [19]. However, these previous reviews were either (i) not systematic [19], (ii) not focused on the youth population, or [18], or (iii) focused exclusively on psychiatric patients [20].

Given recent findings suggesting that NSSI is a separate condition from parasuicidal behaviors, it is relevant to scrutinize previous neuroimaging literature specifically addressing NSSI [21]. Moreover, since the adolescent brain is still developing, youth NSSI’s neural mechanisms possibly differ from those of adults [22]. Therefore, we sought to answer the following research question: What are the findings from neuroimaging studies linking structural and functional abnormalities with NSSI in youth? Our main goal was to systematically review the literature for all neuroimaging studies investigating neural correlates of NSSI, irrespective of imaging modality, that included subjects under 25 years of age.

## 2. Materials and Methods

We performed a systematic review according to a protocol using PRISMA statement guidelines [23]. The review protocol was registered at PROSPERO (registration number: CRD: 42021248254).

### 2.1. Search Strategy

A search strategy was developed using the following terms: (self-injury OR self-directed violence OR self-mutilation OR deliberate self-harm OR nonsuicidal self-injury OR non-suicidal self-injury OR NSSI OR self-harm) AND (computerized tomography OR magnetic resonance imaging OR MRI OR functional magnetic resonance imaging OR fMRI OR positron emission tomography OR single-photon emission computed tomography OR diffusion tensor imaging OR magnetic resonance spectroscopy OR neuroimaging OR gray matter OR white matter). We searched MEDLINE, APA PsycInfo, and Google Scholar for studies published from inception to May 2021. We limited our search to studies reporting on human beings.

### 2.2. PECO Strategy

The construction of the research question followed the PECO strategy [24]:

- Population: samples with a mean age of up to 25 years old
- Exposure: NSSI
- Comparator: healthy control, psychiatric control, or none
- Outcome: neuroimaging (irrespective of the modality)

### 2.3. Screening of Abstracts for Eligibility

Four reviewers (M.J.A.A.B., M.S.C., A.B.R.S., and V.F.D.) independently screened the titles and abstracts. The screening process was conducted in the Rayyan web application [25]. The potentially relevant full texts were read in full by two reviewers (M.J.A.A.B. and M.S.C.), and those that met the inclusion criteria were included. When there was divergence regarding the inclusion of a study, a third reviewer resolved the conflict (P.M.P).

### 2.4. Study Selection

#### 2.4.1. Inclusion Criteria

Eligibility criteria were as follows: (1) participants with NSSI; (2) samples with a mean age of up to 25 years old; (3) studies utilizing neuroimaging methods; (4) study designs with multiple participants (i.e., no case studies); and (5) English as the article language.

#### 2.4.2. Exclusion Criteria for Studies

We excluded preclinical studies, meta-analyses or reviews, and studies that did not include participants with NSSI. 

### 2.5. Data Extraction 

Two reviewers (M.J.A.A.B. and M.S.C.) independently extracted data from included studies. The final decision for inclusion or exclusion of studies in this systematic review was made with reference to the study project registered at PROSPERO. Disagreements about the inclusion or exclusion of the study were resolved by consensus. For each included study, we extracted the following information: author, year, and country of study, number of participants in each group, demographic characteristics of participants (gender and age), study methodology (design, neuroimaging modality, paradigm, and NSSI scale), relevant common confounders (other mental conditions and medication use), and main findings (Table 1). 

### 2.6. Quality Appraisal

The quality of included studies was assessed independently using a version of the Newcastle–Ottawa Scale (NOS) for assessing the quality of nonrandomized studies in meta-analyses for case-control studies [46]. The NOS rates nine items, and when a study meets the criterion for low risk of bias in that item, a star is given. The quality is ranked as unsatisfactory “good” (7–9), “fair” (3–6 stars), or “poor” (0–2 stars). However, as done previously [47], and differently from what was initially proposed by McPheeters and collaborators [48], we adapted the rating by attributing equal weight to each of the nine items, and the total number of stars was used as the cutoff, not the subscore in each of the three domains.

Two open-label studies were evaluated with the Joanna Briggs Institute (JBI) Critical Appraisal Tool for Quasi-Experimental Studies [49], which is a nine-item questionnaire with each question admitting four possible answers—“yes”, “no”, “unclear”, and “not applicable”—and no overall score is calculated.

### 2.7. Data Synthesis and Statistical Analysis 

Relevant aspects of the included studies are individually described in Table 1. The quality assessments are described in Table 2 and Table 3. No meta-analysis could be conducted due to substantial heterogeneity of data.

## 3. Results

### 3.1. Characteristics of Included Studies

**Figure 1:**
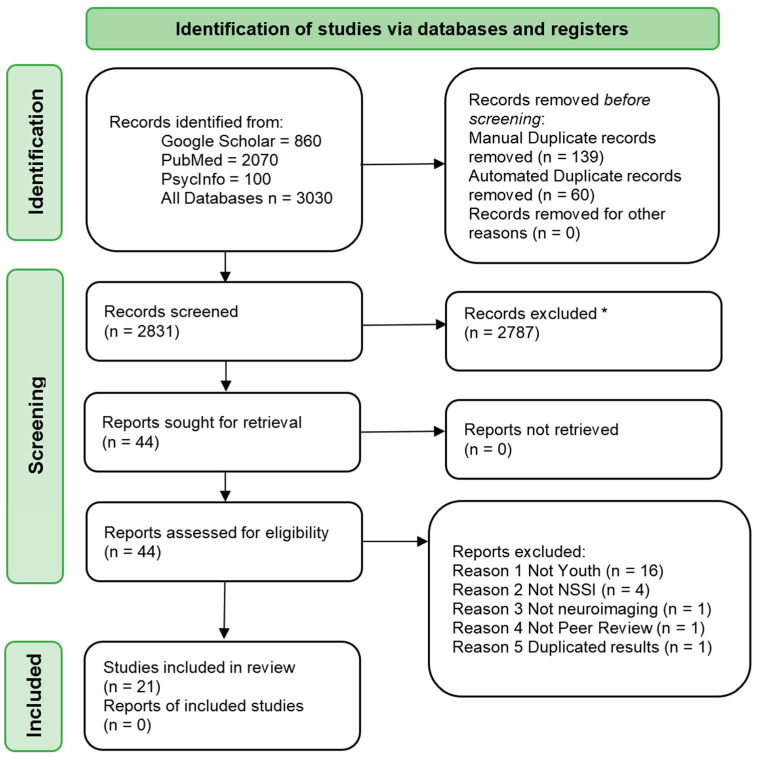
PRISMA Flowchart. * Not using automated methods.

The initial search retrieved 3030 hits, from which 199 were duplicates, and 2787 were removed after evaluations of titles and abstracts (Figure 1). An additional 23 studies were excluded because they did not meet the inclusion criteria. Finally, 21 studies were included in the present review, consisting of a total of 938 subjects.

The sample sizes of the included studies varied from 18–123 individuals. The age range was from 12 to 31 years of age, with a combined mean age and standard deviation of 16.93 (SD 3.47). Most studies included healthy controls as a comparison group (17/21; 80.9%), while a minority (4/21; 19.0%) included controls who fulfilled criteria for a psychiatric disorder. Three studies did not include a control group (3/21; 14.3%). The majority of studies were case-control designs (17/21; 80.9%), while there were two cross-sectional design studies that did not include a control group (2/21; 9.5%). In addition, there were two open-label intervention studies (2/21; 9.5%).

Clinical characteristics of the included samples were diverse, with one study including only BPD patients and another only depressed individuals [26,35]. Psychiatric comorbidity was common among individuals who reported NSSI. Mood, anxiety disorders, and BPD were the most frequent co-occurring disorders (Table 1). Medication use was also prevalent, the most frequent being antidepressant use (Table 1). Several diverse instruments assessed NSSI. The most recurring instruments were the Self-Injurious Thoughts and Behavior Inventory—SITBI (7/21; 33.3%), the Deliberate Self-Harm Inventory —DSHI (4/21; 19.0%), and the Inventory Statements About Self-Injury—ISAS (4/21; 19.0%).

### 3.2. Quality Assessment of Studies

The vast majority of studies (19/21; 90.4%) were classified with an overall quality score of “good” on the NOS Quality Assessment Scale. Only one study (1/21; 4.8%) [29] received a score of “fair”, and one (1/21; 4.8%) was rated as “poor” [31]. Two studies [22,31] lost points in two items of the Selection section of NOS (Definition and Representativeness of cases) due to the exclusive use of advertisements (e.g., online posting, mailing, and flyers) to the local community. In the study by Hooley and collaborators [22], this recruitment strategy did not affect the overall quality score. Although recruiting participants from the community has advantages (e.g., a broader range of severity among cases, with the inclusion of the low severity end of the NSSI spectrum), patients referred by medical institutions are typically seen by other professions, which probably increases the reliability of the NSSI rating. One study [44] used psychiatric controls exclusively, which impacted two criteria of the Selection section (Selection and Definition of controls), but it did not impact the overall quality score. On this particular scale, having psychiatric controls loses points, but this approach can be advantageous in exploring brain mechanisms that are exclusively linked with NSSI rather than psychiatric comorbidity.

Since two studies were open-label trials, we used the JBI Critical Appraisal Checklist for Quasi-Experimental Studies to evaluate their quality. The Cullen et al. (2019) study did not score in two items due to the lack of a comparison group [32]. Santamarina-Perez et al. (2019) receive a “No” in one item due to the fact of acquiring functional MRI (fMRI) data only at baseline [33].

### 3.3. Neuroimaging

Eighteen studies employed fMRI techniques (18/21; 85.7%). Tasks were performed in fifteen of these studies (15/21; 71.4%), in which several paradigms were employed. Most of them explored emotional and interpersonal processes (9/21; 42.8%). Pain, reward, and cognitive processing were also studied. Among the fMRI studies, three studies used only resting-state fMRI (3/21; 14.2%). One study used multimodal both task and resting-state fMRI. Three studies reported on structural MRI methods (3/21; 14.3%).

#### 3.3.1. Structural MRI Findings

The main structural findings involved emotional-processing cortical regions. Two studies suggested diminished insular volumes in NSSI individuals [36,37]. Beauchaine et al. (2018) also found gray matter volume reduction in the right inferior frontal gyrus [37], and Ando et al. (2018) additionally reported that NSSI participants with past suicide attempts had smaller ACC volumes [36]. A tract-based diffusion MRI study found widespread white matter microstructure deficits in cases [28]. Decreased anisotropy in the left cingulum and the left uncinate fasciculus were correlated with, respectively, the time elapsed since the first NSSI episode and higher levels of attentional impulsivity [28]. These findings suggest that among NSSI subjects, greater impulsivity and NSSI severity are associated with significant frontolimbic white matter tract integrity deficits [28].

#### 3.3.2. Resting-State fMRI Findings

Only one study employed a network-based approach and found diminished coherence between the default mode and salience networks and higher connectivity between the central executive and default-mode networks [26]. They also reported an association between higher past-month NSSI and lower coherence in the default mode and insula salience networks [26].

Three studies investigated the connectivity of the amygdala [32,33,38]. Alterations in amygdala–supplementary motor area (SMA) connectivity were differently reported but congruent and complementary between the two studies [32,38]. Cullen et al. (2019) found an association between the reduction in NSSI frequency and lower connectivity between the left amygdala and right SMA, and Schreiner et al. (2017) reported hyperconnectivity between the amygdala and SMA associated with NSSI [32,38]. The first of these studies also found that more significant improvements in self-injury were related to increased connectivity between the right amygdala and right inferior frontal cortex, and the former study also found atypical amygdala–frontal connectivity during resting-state functional connectivity (RSFC). Nevertheless, in the study by Schreiner et al. (2017), this last finding was driven by depressive symptoms [38]. Interestingly, Santamarina-Perez et al. (2019) found that negative amygdala–prefrontal connectivity was associated with greater improvement after the psychotherapeutic intervention of the study [33]. See Table 1 for other main structural findings reported by these studies.

#### 3.3.3. Task-Based fMRI Findings

##### Pain and Aversive Stimuli Processing

There were three task-based fMRI studies involving unpleasant physical sensations and pain processing. Osuch et al. (2014) used a painfully “cold” and “cool” stimuli comparator condition, either self- or experimenter-administered [44]. It was demonstrated that NSSI participants had greater activity than psychiatric controls in areas related to pain processing and dopaminergic systems, such as the right midbrain, pons, and amygdala. In addition, another region—including the orbitofrontal cortex (OFC)—related to reward processing, and the opiate system was hyperactivated in NSSI subjects. Surprisingly, there was no interaction between pain and neural activity. However, there was a correlation between relief in the self-administered cold condition and several areas, including subcortical regions (right thalamus, caudate, globus pallidus) and a large area involving the precuneus, right supramarginal gyrus, cingulate, occipital, and angular gyrus. Lastly, it was observed across the paradigm conditions less connectivity between OFC and ACC.

One study applied a dose-level approach to pain stimuli [43]. Findings suggested that individuals reporting previous NSSI behavior did not modulate the activation of the anterior insula as the unpleasant stimuli increased. Previous studies implicated this region in affective aspects of the processing of aversive sensations [50]. On the other hand, modulation of the posterior insula, associated with the discrimination of unpleasant stimuli [51], and the somatosensory cortex were intact. In a study with BPD self-injurers and young, healthy controls using the same paradigm of scalable unpleasant electric stimuli, researchers found intensity encoding activation within the primary and secondary somatosensory cortex, posterior insula, posterior midcingulate cortex (pMCC), and SMA [35]. However, there were no differences among groups. Moreover, there was a lack of activation of pain processing networks associated with the affective components of pain. The last finding may be related to the widespread use of antidepressants, which might have caused an affective regulation effect [52].

##### Reward Processing

Two studies employed monetary incentive-based tasks. Sauder et al. (2016) analyzed data from an adolescent female sample using the monetary incentive delay task. NSSI behavior was associated with lower activation in striatal, amygdala, and orbitofrontal regions during the anticipation of the reward [42]. On the other hand, Poon et al. (2019) found increased bilateral activation of dorsal reward-related regions (putamen) related to NSSI ideation (a proxy for future risk of NSSI) in response to reward outcome in early adolescents. However, there are limitations in this conclusion due to the lack of a control group [31].

The study by Hooley et al. (2020), described below, also evaluated regions involved in reward processing (nucleus accumbens and ventral tegmental area) using an emotional elicitation task but found no activation of this reward circuitry. However, in NSSI participants, significantly greater activation was found in the OFC [22], a region involved in encoding the value of reward representation [53].

##### Emotional Processing

Five studies evaluated how NSSI patients process emotional stimuli [22,27,29,38,45]. Among these, three studies utilized affective and NSSI-related images to elicit emotional responses [22,27,45]. Plener et al. (2012) found hyperactivity of the limbic region (amygdala, hippocampus) and ACC when adolescents with NSSI visualized emotional pictures, though depression explained the differences between patients and controls. Other areas (middle orbitofrontal and inferior/middle frontal cortex) were more sensitive to NSSI-related pictures [45]. Mayo et al. (2020) found activation of amygdala, occipital, and frontal regions, but no difference between the groups. However, they found an interesting association between an objective measure of emotion reactivity (eletroneuromiography of zygomatic and corrugator muscles) and anterior insula activation [27]. In the study by Hooley et al. (2020), the healthy comparison group showed greater amygdalar activation in response to NSSI and negative pictures, while the young adult NSSI group showed an increase in the cingulate cortex (CC) and OFC activation for the same pictures. The same study found greater activation in the amygdala and OFC for positive pictures in the NSSI group [22].

Researchers also studied emotional processing using responses to facial expressions. Demers et al. (2019) had studied automatic emotion processing (opposed to conscious awareness of emotions) using a masked emotion face task. They found that externally oriented thinking (EOT), a facet of alexithymia that relates to the difficulty in attending to emotions, was related to decreased activation to masked happy faces in the right frontal gyrus, right supramarginal gyrus (SMG), and left precentral gyrus among NSSI female [29]. Moreover, EOT was associated with increased activation to masked fear (relative to fixation) in the right SMG. In addition to the already mentioned RSFC study, Schreiner et al. (2017) also analyzed functional connectivity (i.e., task functional connectivity—TFC) during an emotional face-matching task. This study demonstrated an even more prominent amygdala–frontal hypoconnectivity than observed in the RSFC, which did not hold after controlling for depression. Additionally, NSSI had positive connectivity between the amygdala and regions of the occipital cortex [38].

##### Interpersonal and Self-Processing

The Cyberball task is a well-known paradigm that elicits feelings related to social inclusion and exclusion [54]. This paradigm was employed in two studies. One of them showed that during the exclusion phase, when subjects experienced rejection, depressed individuals with NSSI showed enhanced activation of the medial prefrontal cortex (mPFC) and the ventrolateral prefrontal cortex (vlPFC) when compared with non-NSSI depressed and healthy adolescents [40]. Another study showed that NSSI adolescents without BPD had activation of putamen during the exclusion phase [39]. Enhanced activation in the ventral anterior cingulate during social exclusion was observed both in BPD and NSSI subjects.

Using a novel simulated online game paradigm in which participants judged a picture of others or were judged by simulated players, Perini et al. (2019) showed that brain regions—including the subgenual anterior cingulate cortex (sgACC), the dorsomedial prefrontal cortex (dmPFC), and the PCC—significantly contributed to the discrimination of NSSI from healthy controls. However, there was no difference between activity in the right anterior insula and dorsal anterior cingulate cortex (dACC), regions that are crucial nodes of the salience networks [30].

Finally, one study investigated the neural encoding of self-processing through an interpersonal self-processing task in which participants listened to statements about themselves and significant others. NSSI youth showed greater limbic and anterior and posterior cortical midline structures across all perspectives when compared with depressed and healthy controls. In addition, NSSI demonstrated higher limbic (amygdala, hippocampus, and parahippocampus) and fusiform activation when self-processing their mother’s perspective about them. When processing their classmates’ points of view, greater activity was observed in the precuneus and PCC in NSSI youth [41].

##### Executive Function Processing

Only one study employed a cognitive interference task, a component of executive function associated with attentional control and inhibitory processing [55]. Dahlgren et al. (2018) demonstrated a different activation pattern of the cingulo-frontal-parietal (CFP) attentional network circuitry during the Multi-Source Interference Task (MSIT). The NSSI group showed an increased cingulate cortex (CC) and a decreased dorsolateral cortex (dlPFC) activation, although no differences in objective performance were observed. Moreover, a decreased activation in dlPFC was correlated with self-reported impulsivity and emotion reactivity in the NSSI sample [34]. This different neural pattern may represent a compensatory activation of the CC due to impaired moment-to-moment processing during the MSIT [34].

## 4. Discussion

We systematically reviewed the literature on youth NSSI and its neural correlates. Alterations in potential circuits involved in pain, reward, emotional, interpersonal, self, and executive function processes were identified. Although consistent results were found among studies, the diversity of neuroimaging methods (e.g., modality, paradigms, regions under study) makes comparisons challenging. Additionally, we found different methodological limitations: samples were predominantly female, clinical groups were psychopathological and heterogeneous, a few studies lacked a control group, NSSI instruments varied considerably, no study had a significant longitudinal follow-up. In addition, suicidal behavior and depressive symptoms were common in patients, making it difficult to separate which alterations were specific to NSSI. Many studies reported medication use, but the vast majority did not report psychotherapy treatment, which is the first-line treatment for NSSI [56]. Moreover, it is well established that psychotherapeutic outcomes are associated with changes in brain function and structure [57]. Furthermore, only one study reported mainly negative findings, which is a valuable practice to mitigate positive publishing bias [35]. Several studies included in this review found anomalies in cortico-limbic structures that are involved in emotional processing. The amygdala—a region that is sensitive to threat and fear [58]—was shown to be hyperactivated to emotional and NSSI pictures in NSSI adolescents [45]. Increased activation of limbic structures, such as the amygdala, hippocampus, and parahippocampus, were also found during an interpersonal self-processing task [41]. On the other hand, Hooley et al. (2020) found decreased amygdala activation to negative and NSSI images and increased activation to positive images. One possible interpretation is that NSSI becomes less aversive over time through a process of classical conditioning [22]. We also identified studies that showed an increase in ACC and OFC activity during emotional processing tasks [22,45]. The ACC connects limbic and cognitive systems [59], and the OFC is associated with the attribution of value to emotional stimuli [60]. A possible explanation is that these cortical regions could be compensating for the overactivation of limbic structures [45]. Functional connectivity studies also suggested an atypical amygdala–frontal integration in emotional face matching tasks and a baseline predictor of psychotherapy efficacy [33,38]. Additionally, higher connectivity between the amygdala in the inferior frontal cortex was associated with greater improvement in NSSI after treatment [32]. These alterations corroborate the hypothesis that individuals with NSSI have atypical emotional processing at the neurobiological level and may process limbic hyperreactivity through different cortical modulation systems, which may result from the lack of “top-down” prefrontal regulation. This model is corroborated by a study that showed a different pattern activation of the CFP attentional network circuitry involved in inhibitory processes. Specifically, a decrease in the dlPFC activation correlated with emotional control and impulsivity [34]. NSSI could be a coping strategy among youth who lack more sophisticated skills for which good cortical–limbic integration is required. Fortunately, this is probably amenable to psychotherapeutic treatment [33]. Additionally, some individuals may lose aversion to NSSI stimuli over time, removing a natural barrier to self-injury [61].

The insula, a component of the salience network and closely related to the limbic system [62], was found to be altered in individuals with NSSI [26,27,33,36,37,43]. This region is responsible for encoding negative emotions, attention modulation, interoception, and pain perception [63]. In addition to volumetric reduction [36,37], anterior insula activation was correlated with objective emotional reactivity in female adolescents [27]. In this study, electromyography objectively identified a greater intensity of facial expressions in the NSSI group, despite no differences in self-reported affect, suggesting a suppression or avoidance of the emotional response [27]. Moreover, limited emotion awareness was associated with altered facial mask processing [29] and interoceptive awareness deficits with diminished coherence in the default mode and the insula salience networks [26]. The link between dissociation and NSSI is well established in the literature [64]. When taken together, the above findings could also be interpreted as neurobiological correlates of its psychopathological manifestation. In various mental conditions, the co-occurrence of dissociation can complicate treatment even further. For example, evidence-based treatments for emotional dysregulation and trauma routinely use anti-dissociation skills to decrease emotional arousal and maximize learning [65,66] since it can reduce treatment therapy efficacy [67]. Therefore, identifying dissociative symptoms in NSSI patients is probably wise and clinically relevant.

Only a few studies focused on the reward system [31,42], which is also implicated in affective regulation [60]. One study showed that NSSI had a blunted response in striatal and OFC regions in a situation of anticipation of monetary reward [42]. This mesolimbic dysfunction predisposes to impulsive behavior, irritability, and depression that frequently co-occur in self-injury individuals [2,68]. Another study [31] found an association of heightened putamen activation and NSSI ideation, a proxy for future risk for NSSI, among early adolescents during monetary reward. Reward hypersensitivity may lead to risky behavior among youths [69]. A recent longitudinal self-harm (not exclusively NSSI) study showed different pathways to self-harm: one of them is characterized by higher psychopathology levels, and the other is characterized by risky youth behavior [70].

Often, affective instability in NSSI patients occurs in interpersonal contexts when teens experience criticism or rejection [71]. Some paradigms were specifically designed to simulate these contexts. During the Cyberball task, there was a relative increase in the activation of mPFC, vlPFC, and putamen during the social exclusion phase [39,40]. The activation of mPFC is associated with mentalizing, and vlPFC is associated with the experience of social exclusion, regulation of affect, and pain [40]. The putamen is part of the salience network [72]. In conjunction, these findings could corroborate the clinical impression that these adolescents are more sensitive to social cues and ruminate more about the reasons why they were rejected. In mentalization terms, hypermentalizing—originally defined in BPD—is a process in which adolescents compulsively think about other people’s thoughts and feelings [5,73]. Regions of the posterior cortical midline are also implicated in interpersonal processes in NSSI adolescents. When processing peers’ perspectives about the self, NSSI showed greater activity in the PCC and precuneus activity, regions related to processing information about the self [41]. Additionally, during a self-evaluative online game, activity in PCC, sgACC, and dmPFC differentiated the NSSI from the healthy control group. Authors suggest that self-referential social processes and autobiographical memory may play a role contributing to a negative interpretation bias that leads NSSI adolescents to feel more rejected, perhaps recalling negative memories regarding their peers [30,41]. The sgACC is also associated with mood disorders [74].

Individuals with NSSI might also have atypical neural pain processing systems [35,43,44]. The somatosensory-discriminative circuitry of pain, including regions of the primary and secondary somatosensory cortex, the posterior insula, and the SMA, was unimpaired [35]. However, affective pain processing involving the anterior insula was altered in one study [43]. Another aspect relating to pain processing is the overlap between aversive stimuli processing and reward systems in NSSI. An association of relief during self-administered painfully cold stimuli was demonstrated [44]. NSSI subjects may have higher activation in the traditional limbic dopaminergic system (right midbrain and pons) and cognitive processing region associated with hedonic experience (OFC) [44]. These preliminary findings suggest that pain processing may be one of the factors related to self-injury, as proposed by several models [1,61,75].

Finally, it is important to highlight that several neurodevelopmental processes begin very early in life (e.g., gestational period and first 1000 days) [76]. Moreover, brain maturation does not occur uniformly across distinct brain regions. For instance, from the “brain connectome” perspective, there is a developmental shift from short-range connections in childhood to a broader long-range integration of neural regions in adolescence [77,78]. Subcortical functioning also seems to outpace the maturation of frontal cortical regions in adolescence, and this asynchrony may differently influence vulnerability to NSSI in this age range. Altogether, these neurodevelopmental mechanisms can influence neuroimaging findings in youth samples, which has not been completely addressed in NSSI studies. Additionally, environmental factors may influence brain development through mechanisms such as gene expression and epigenetics (Gene x Environment interactions) [76]. Since stressful life events are risk factors for both NSSI and atypical cortical maturation, futures studies must consider this potential confounding factor when interpreting neuroimaging findings.

## 5. Conclusions

This systematic review summarized neuroimaging alterations from twenty-one studies on youth with NSSI. Several findings from specific neural circuitries were associated with self-injury’s neural processes, e.g., pain, reward, emotional, interpersonal, self, and inhibitory control processing. Thus, NSSI seems to have complex neurobiological underpinnings involving multiple subcortical and cortical regions. From a theoretical point of view, the nature of NSSI is multifactorial, and this review supports this idea. Future studies, then, should focus on longitudinal designs with different sources of variability (e.g., neuroimaging, serum markers, self-report, behavioral paradigms) to shed light on the interactions among these different factors.

## Figures and Tables

**Table 1:** Characteristics of included studies.

Author, Year	N (F) NSSI/Controls	Age (Mean ± SD) NSSI/Controls	Study Design	Imaging Modality	Paradigm	NSSI Scale	Mental Conditions	Medications	Main Findings
Ho et al. 2021 [26]	29 (20F)/21 (12F)	16.34 ± 1.37/16.15 ± 1.29	Case-control, HC	fMRI/whole brain	Resting-state	SITBI	MDD 100%, suicidal ideation 69%, suicide attempt 38%	48% on medications	↓ coherence in the ventral and anterior default mode and the insula salience networks / ↑ connectivity between the central executive and the default mode networks / ↓ coherence in the default mode and the insula salience networks was associated with higher past-month NSSI
Mayo et al. 2020 [27]	30 (30F)/30 (30F)	15.9 ± 0.79/16.4 ± 1.00	Case-control, HC	fMRI/whole brain	Affective and NSSI-related pictures task	CANDI	Lifetime suicidal ideation 100%, lifetime suicide attempt 36.7%, MDD 50.0%, ADHD 50.0%, BPD 43.3%, social anxiety disorder 30.0%, autism spectrum disorder (high functioning) 13.3%, panic disorder 10.0%, eating disorder (unspecified) 10.0%, ODD/CD 10.0%, GAD 6.67%, atypical anorexia nervosa 6.67%, agoraphobia 3.30%, anxiety disorder (unspecified) 3.30%, PTSD 3.30%, anorexia nervosa 3.30%	No medication 63.3%, SSRI/SNRI 26.7%, SSRI/SNRI + methylphenidate 3.30%, antipsychotic 3.30%, SSRI/SNRI + neuroleptic 3.30%	No differences in self-reported affect during task / ↑ positive (e.g., zygomatic) and negative (e.g., corrugator) reactivity during task / + correlation between anterior insula response and the averaged electromyography magnitude
Hooley et al. 2020 [22]	15 (15F)/15 (15F)	21.27 ± 3.67/22.80 ± 3.28	Case-control, HC	fMRI/ROI /amygdala, CC, OFC, NAcc, and VTA	Affective and NSSI-related pictures task	SITBI	BPD 86.67%, mood disorders 80.00%, anxiety disorders 53.33%, eating disorders 6.67%, past alcohol dependence 6.67%, suicide attempt 26.67%	NA	↓ amygdala and ↑ CC and OFC activation to NSSI and negative images / ↑ amygdala and OFC activation to positive images
Schreiner et al. 2020 [28]	28 (28F)/22 (22F)	17.53 ± 2.36/17.69 ± 2.26	Case-control, HC	Diffusion MRI/structural (white matter (*TBSS*)/whole brain	—	DSHI, ISAS	MDD 57%, GAD 29%, depression NOS 18%, PTSD 18%, Specific Phobia 11%, panic disorder 11%, anxiety disorder NOS 7%, OCD 7%, ADHD 7%, alcohol dependence 7%, social phobia 4%, eating disorder NOS 4%, no current disorder 18%	Currently medicated 43%, antidepressants 32%, stimulants 7%, antipsychotics 4%, anxiolytics/benzodiazepines 14%, other psychotropics 4%	↓ generalized fractional anisotropy (GFA) in several white matter tracts, including the uncinate fasciculus, cingulum, bilateral superior and inferior longitudinal fasciculi, anterior thalamic radiation, callosal body, and corticospinal tract / ↓ GFA in the left cingulum was associated with NSSI duration / ↓ GFA in the left uncinate fasciculus was associated with higher levels of attentional impulsivity
Demers et al. 2019 [29]	25 (25F)/No controls	17.30 ± 2.35/No controls	Cross-sectional, No controls	fMRI/whole brain	Masked emotional facial expressions	DSHI, ISAS	Mood disorder 84%, anxiety disorder 48%, alcohol use disorder 4%, eating disorder 12.5%, PTSD 4%	Almost half the participants on medications (no specification)	Externally-oriented thinking, a facet of alexithymia, was related to differential reactivity to masked emotional faces in clusters in the right supramarginal and right inferior frontal gyri
Perini et al. 2019 [30]	30 (30F)/30 (30F)	15.9 ± 0.79/16.4 ± 1.00	Case-control, HC	fMRI/whole brain	Simulated online game	CANDI	Suicidal ideation 100%, suicidal attempt 36.7%, MDD 50.0%, anxiety disorder 43.3%, PTSD 3.3%, borderline traits 43.3%, eating disorder 20.0%, ADHD 50.0%, autism (high functioning13.3%), ODD/CD 10.0%	SSRI/SNRI 26.7%, SSRI/SNRI + methylphenidate 3.3%, neuroleptic 3.3%, SSRI/SNRI + neuroleptic 3.3%, no medication 63.3%	Negative bias in processing social feedback from others / Brain regions that classified NSSI subjects and controls included dmPFC and sgACC cortices
Poon et al. 2019 [31]	71 (33F)/No controls	12.56 ± 0.65/No controls	Cross-sectional, No controls	fMRI/ROI/caudate, putamen, NAcc, vmPFC	Monetary reward	SITBI	No diagnosis 94.4%, MDD 2.8%, GAD 1.4%, ODD 1.4%	Medication use was an exclusion criterion.	↑ activation in the bilateral putamen associated with NSSI thoughts
Cullen et al. 2019 [32]	18 (18F)/No controls	17.11 ± 2.07/No controls	Open-label trial, No Controls	fMRI/ROI/amygdala and the nucleus accumbens	Resting-state	DSHI, ISAS	NA	N-acetylcysteine 100%, other medications 22%	Reduction in NSSI frequency was associated with: ↓ in left amygdala connectivity between right SMA / ↑ in right amygdala connectivity between right inferior frontal cortex / ↓ in connectivity between right nucleus accumbens and left superior medial frontal cortex
Santamarina-Perez et al. 2019 [33]	24 (21F)/16 (13F)	15.42 ± 0.97/15.50 ± 1.10	Open-label trial, HC	fMRI/ROI/amygdala and the mPFC	Resting-state	C-SSRS (specific items), SIQ-Jr	MDD 87%, anxiety disorder (any) 46%, eating disorder 33%, PTSD 29%, bipolar disorder 12,5%, PTSD 29%	Antipsychotics 75%, antidepressants 63%, lithium 8%	↓ connectivity between the amygdala and the ACC, subcallosal cortex, and paracingulate gyrus / ↓ connectivity between the amygdala and the right planum temporale and right insula / ↓ connectivity between the mPFC and the precentral and postcentral gyri and the left insula / Stronger negative amygdala–prefrontal connectivity was associated with greater posttreatment improvement in NSSI / Greater positive baseline amygdala–brainstem connectivity was associated with NSSI improvement
Dahlgren et al. 2018 [34]	15 (15F)/15 (15F)	21.27 ± 3.67/22.80 ± 3.28	Case-control, HC	fMRI/ROI/CC and dlPFC	Multi-source interference task	SITBI	BPD 86.7%, mood disorders 80.0%, anxiety disorders 53.34%, eating disorders 6.7%, alcohol dependence 6.7%, suicide attempt 26.67%	Antidepressants 16.6%, antipsychotics 6.7%, anxiolytics 6.7%, stimulants 3.34%	↑ activation in CC and ↓ activation in dlPFC during task / dlPFC activation inversely correlated with emotional reactivity and impulsivity
Malejko et al. 2018 [35]	15 (15F) (BPD)/15 (15F) (HC)	23.33 ± 1.07/23.27 ± 1.11	Case-control, HC	fMRI/whole brain	Unpleasant (but not painful) electric stimulation	FASM	BPD 100%, MDD 86.7%, PTSD 53.3%, dysthymia 13.3%	Antidepressants 86.7%, lithium 6.7%	Significant intensity-encoding neural activations were observed within the primary and secondary somatosensory cortex, the posterior insula, the posterior midcingulate cortex, and SMA in both HC and BPD / No significant between-group differences in intensity-encoding neural activations, even at lowered significance thresholds
Ando et al. 2018 [36]	29 (29F)/21 (21F)	15.9 ± 1.3/15.8 ± 1.1	Case-control, HC	MRI (structural)/volume (*Freesurfer*)/ROI/lateral and medial PFC, OFC, ACC, insula, thalamus, hippocampus, and amygdala	—	SITBI	Mood disorders 82.8%; neurotic, stress-related and somatoform disorders 58.6%; suicide attempt 55.2%, BPD 45%; behavioral and emotional disorders with onset in childhood and adolescence 31.0%; mental and behavioral disorders due to psychoactive substance use 27.6%; behavioral syndromes associated with physiological disturbances and physical factors 17.2%	NA	↓ regional grey matter volume in insula / Suggestion (not survived correction for multiple comparisons) of ↓volume in ACC / Even smaller ACC volume in adolescents engaging in NSSI with a history of suicide attempt in comparison to those with no history of suicide attempt
Beauchaine et al. 2018 [37]	20 (20F)/20 (20F)	15.70 ± 1.77/15.93 ± 2.03	Case-control, HC	MRI (structural)/volume (VBM)/whole brain	—	L-SASI	1.25 suicide attempts, 3 symptoms of BPD (averages)	NA	↓ gray matter volumes in the insular cortex bilaterally and in the right inferior frontal gyrus / Insular and inferior frontal gyrus gray matter volumes correlated inversely with self-reported emotion dysregulation, over-and-above effects of psychopathology.
Schreiner at al. 2017 [38]	25 (25F)/20 (20F) (RSFC); 24 (24F)/17(17F) (Task)	17.57 ± 2.49/18.01 ± 2.08 (RSFC) 17.34 ± 2.44/17.98 ± 2.00 (Task)	Case-control, HC	fMRI/ROI/amygdala	Resting-state and emotion face-matching functional connectivity	DSHI, ISAS	RSFC and task, respectively: MDD 52%, 58%; depressive disorder NOS 20%, 13%; GAD 24%, 21%; anxiety disorder NOS 4%, 8%; social phobia 4%, 8%; specific phobia 12%, 8%; panic disorder (8%) (8%; PTSD 12%, 13%; OCD 8%, 4%; eating disorder NOS 4%, 4%; ADHD 4%, 4%; alcohol dependence 8%, 4%; no current disorder 20%, 21%	RSFC and task data, respectively: currently medicated 42%, 44%; antidepressants 29%, 35%; stimulants 4%,4%; antipsychotics 4%, 4%; anxiolytics/benzodiazepines 13%, 13%; other psychotropics 4%, 4%	Atypical amygdala–frontal connectivity driven by depression symptoms (RSFC and task) / Hyperconnectivity between amygdala and SMA independent of depression symptoms (RSFC) / Widespread amygdala–cortical connectivity anomalies (RSFC and task)
Brown et al. 2017 [39]	NSSI: 13 (10F) NSSI and BPD: 14 (14F) HC (adolescents): 15 (12F) HC (young adults): 17 (17F)	NSSI: 15.5 ± 2.0 NSSI and BPD: 23.6 ± 4.1 HC (adolescents): 14.5 ± 1.7 HC (young adults): 23.2 ± 4.4	Case-control, HC, and Psychiatric Controls	fMRI/whole brain	“Cyberball”	SITBI	NSSI and BPD, respectively: major depression 100%, 100%; hyperkinetic disorder 23%, 0; eating disorder 15%, 7%; anxiety disorder 15%, 14%; PTSD 0, 50%	NSSI and BPD, respectively: antidepressants 15%, 86%; mood stabilizers 0, 7%	NSSI and BPD showed enhanced feelings of social exclusion as compared with HC / ↑ activation in the putamen during social exclusion versus inclusion in NSSI / ↑ activation in the ventral anterior CC during social exclusion in NSSI and BPD / ↑ activation in DLPFC, dmPFC, and the anterior insula during social inclusion as compared with a passive watching condition in BPD
Groschwitz et al. 2016 [40]	NSSI and depression: 14 (11F) Depression: 14 (11F) HC: 15 (12F)	NSSI and depression: 15.4 ± 1.9 Depression: 15.9 ± 1.6 HC: 14.5 ± 1.7	Case-control, HC, and Psychiatric Controls	fMRI/whole brain	“Cyberball”	SITBI	NSSI and depression, and depression, respectively: major depression 100%, 100%; anxiety disorder 7%, 14%; PTSD 0, 29%; eating disorder 14%, 21%; ADHD 14%, 7%; conduct disorder 21%, 7%	NSSI and depression, respectively: antidepressants 14%, 50%; psychostimulants 7%, 0	↑ activation of the mPFC and the VLPFC in depressed adolescents with NSSI compared with depressed adolescents without NSSI and also compared with HC
Quevedo et al. 2016 [41]	NSSI: 50 (32F) Depression: 36 (17F) HC: 37 (18F)	NSSI: 14.94 ± 1.54 Depression: 14.77 ± 1.86 HC: 14.49 ± 1.53	Case-control, HC, and Psychiatric Controls	fMRI/whole brain	Interpersonal self-processing task	K-SADS (item)	Depression: 100% in both clinical groups suicide ideation: NSSI > depression group	HC, depression and NSSI, respectively: antidepressants 0, 33.3%, 48%; antipsychotic 0, 0, 12%; mood stabilizers 5%, 0, 2%; stimulants 0, 11%, 12%; anxiolytic 0, 3%, 10%	↑ activation in limbic areas, and anterior and posterior cortical midline structures in NSSI versus DEP and HC / ↑ activity in rostrolateral, frontal pole, and occipital cortex in HC versus NSSI and DEP / ↑ responses in amygdala, hippocampus, parahippocampus, and fusiform in NSSI when taking their mother’s perspective, which was negatively correlated with self-reports of the mother’s support of adolescent’s emotional distress in the NSSI group / ↑ precuneus and posterior cingulate cortex activity in NSSI during indirect self-processing from their classmates’ perspective
Sauder et al. 2016 [42]	19 (19F)/19 (19F)	15.93 ± 2.03/15.70 ± 1.77	Case-control, HC	fMRI/ROI/striatum (caudate + putamen) and OFC	Monetary incentive delay task	L-SASI	Depression 47%, substance use disorder 16%	SSRI 26%	↓ activation in striatal and OFC regions during anticipation of reward / ↓ bilateral amygdala activation during reward anticipation
Bonenberger et al. 2015 [43]	14 (14F)/16 (16F)	21.1 ± 2.51/23.4 ± 3.72	Case-control, HC	fMRI/ROI/insula and somatosensory cortex	Unpleasant haptic electric stimulation	SITBI	BPD 21.4%, MDD 14.2%, agoraphobia 14.2%	No medication	Activation of the anterior insula was significantly modulated only in HC, but not in subjects with NSSI
Osuch et al. 2014 [44]	NSSI: 13 (10F) Non-NSSI: 15 (13F)	NSSI: 20 ± 2.4 Non-NSSI: 21 ± 1.8	Case-control, Psychiatric Controls	fMRI/whole brain	Painfully “cold” and “cool” stimulus	SIMSv2; OSI	NSSI and non-NSSI, respectively: MDD 46%, 27%; bipolar type I 15%, 13%; bipolar type II 8%, 0; OCD 15%, 7%; GAD 8%, 20%; panic disorder 15%, 13%; social phobia 31%, 27%; specific phobia 15%, 7%; hypochondriasis 8%, 7%; PTSD 23%, 0; bulimia 0, 7%; adjustment disorder (past) 0, 7%; alcohol abuse 0, 7%; alcohol dependence 8%, 7%; drug abuse 15%, 13%; acute stress 0, 7%	NSSI: antidepressants (61.5%); mood stabilizer (7.7%); atypical antipsychotic (7.7%) Non-NSSI: antidepressants (53.3%); mood stabilizer (6.7%); MAO+modafinil (6.7%); benzodiazepine (6.7%)	↑ activation in right midbrain/pons, culmen, amygdala, OFC and parahippocampal, inferior frontal and superior temporal gyri in NSSI / ↑ activation associated with a subjective sense of “relief” in areas associated with reward/pain and addiction, including thalamus, dorsal striatum, and anterior precuneus / ↓ functional connectivity between right OFC and anterior CC in NSSI youth (post hoc analysis)
Plener et al. 2012 [45]	9 (9F)/9 (9F)	15.2 ± 1.5/15.0± 0.9	Case-control, HC	fMRI/whole brain	Affective and NSSI-related pictures task	OSI; FASM; SHBQ	MDD 66.7%, suicide attempt 54%, mild depression disorder 11.1%, dysthymia 11.1%, PTSD 11.1%, BPD 22.2%, combined personality disorder 11.1%	Antidepressant 11.1%	NSSI group rated pictures with self-injurious reference as significantly more arousing than controls / ↑ activation in amygdala, hippocampus, and anterior cingulate cortex bilaterally during emotional pictures / Depression explained differences between groups in the limbic area / ↑ activation in the middle OFC, and inferior and middle frontal cortex during NSSI pictures / ↓ activation in the occipital cortex in correlation to arousal and inferior frontal cortex in correlation to valence when watching emotional pictures

If not otherwise specified, all information on columns pertains to the NSSI group. *Abbreviations: Scales:* SITBI, Self-Injurious Thoughts, and Behaviors Interview; CANDI, Clinical Assessment of Nonsuicidal Self-Injury Disorder; ISAS, Self-Report Inventory of Statements About Self-Injury; DSHI, Deliberate Self-Harm Inventory; C-SSRS, Columbia Suicide Severity Rating Scale; SIQ-Jr, Suicidal Ideation Questionnaire-Junior; FASM, Functional Assessment of Self-Mutilation; L-SASI, Lifetime Suicide Attempt, and Self-Injury Interview; OSI, Ottawa Self-Injury Inventory; SIMSv2, Self-Injury Motivation Scale v.2; K-SADS, Schedule for Affective Disorders and Schizophrenia for School-Aged Children; SHBQ, Self-Harm Behaviour Questionnaire. *Brain regions:* CC, cingulate cortex; OFC, orbitofrontal cortex; SMA, supplementary motor area; ACC, anterior cingulate cortex; mPFC, medial prefrontal cortex; dlPFC, dorso lateral prefrontal cortex; VLPFC, ventrolateral prefrontal cortex; vmPFC, ventromedial prefrontal cortex; VTA, ventral tegmental area; NAcc, nucleus accumbens; sgACC, subgenual anterior cingulate cortex; dmPFC, dorsomedial prefrontal cortex. *Neuroimaging methods: MRI, magnetic resonance imaging; fMRI, functional MRI;* VBM, voxel-based morphometry; RSFC, resting-state functional connectivity.

**Table 2:** Quality of included studies evaluated with Newcastle–Ottawa Scale for quality assessment of case-control studies.

	Selection				Comparability	Exposure				
Author, Year	1 Is the Case Definition Adequate?	2 Representativeness of the Cases	3 Selection of Controls	4 Definition of Controls	1 Comparability of Cases and Controls on the Basis of the Design or Analysis	1 Ascertainment of Exposure	2 Same Method of Ascertainment for Cases and Controls	3 Non-Response Rate	TOTAL STARS (0–9)	Quality Overall Score
Ho et al. 2021 [26]	*	*	*	*	**	*	*	*	9	good
Mayo et al. 2020 [27]	*	*	*	*	**	*	*	◯	8	good
Hooley et al. 2020 [22]	◯	◯	*	*	**	*	*	*	7	good
Schreiner et al. 2020 [28]	*	*	*	*	**	*	*	*	9	good
Demers et al. 2019 [29]	*	*	◯	◯	◯	*	◯	*	4	fair
Perini et al. 2019 [30]	*	*	*	*	**	*	*	*	9	good
Poon et al. 2019 [31]	◯	◯	◯	◯	◯	*	◯	*	2	poor
Dahlgren et al. 2018 [34]	*	*	*	*	**	*	*	*	9	good
Malejko et al. 2018 [35]	*	*	*	*	**	*	*	*	9	good
Ando et al. 2018 [36]	*	*	*	*	**	*	*	◯	8	good
Beauchaine et al. 2018 [37]	*	*	*	*	**	*	*	*	9	good
Schreiner et al. 2017 [38]	*	*	*	*	**	*	*	*	9	good
Brown et al. 2017 [39]	*	*	*	*	**	*	*	◯	8	good
Groschwitz et al. 2016 [40]	*	*	*	*	**	*	*	◯	8	good
Quevedo et al. 2016 [41]	*	*	*	*	**	*	*	◯	8	good
Sauder et al. 2016 [42]	*	*	*	*	**	*	*	*	9	good
Bonenberger et al. 2015 [43]	*	*	*	*	**	*	*	◯	8	good
Osuch et al. 2014 [44]	*	*	◯	◯	**	*	*	*	7	good
Plener et al. 2012 [45]	*	*	*	*	**	*	*	◯	9	good

*Quality Score: “good” (7–9 stars), “fair” (3–6 stars), or “poor” (0–2 stars).* *, A study can be awarded a maximum of one star for each item within the Selection and Exposure categories. **, A maximum of two stars can be given for Comparability. ◯, The circle means “no star”.

**Table 3:** Quality of included studies assessed by the JBI Critical Appraisal Checklist for Quasi-Experimental Studies.

	Author, Year	
Questions	Cullen et al. 2019 [32]	Santamarina-Perez et al. 2019 [33]
1. Is it clear in the study what is the “cause” and what is the “effect” (i.e., there is no confusion about which variable comes first)?	Yes	Yes
2. Were the participants included in any comparisons similar?	Yes	Yes
3. Were the participants included in any comparisons receiving similar treatment/care, other than the exposure or intervention of interest?	Not Applicable	Yes
4. Was there a control group?	No	Yes
5. Were there multiple measurements of the outcome, both pre and post the intervention/exposure?	Yes *	No **
6. Was follow-up complete, and if not, were differences between groups in terms of their follow-up adequately described and analyzed?	Yes	Yes
7. Were the outcomes of participants included in any comparisons measured in the same way?	Yes	Yes
8. Were outcomes measured in a reliable way?	Yes	Yes
9. Was appropriate statistical analysis used?	Yes	Yes
Total	7	8

* Neuroimaging performed in one-time point before intervention and in one-time point after the intervention; ** Only baseline neuroimaging.

## Data Availability

Data is contained within the article.
